# Measles, mistrust, and missed protection in the Americas

**DOI:** 10.1016/j.lana.2026.101504

**Published:** 2026-05-15

**Authors:** The Lancet Regional Health – Americas

Measles cases are escalating across the Americas, exposing a convergence of immunisation gaps, inequitable access, vaccine hesitancy, misinformation, and weakened institutional trust. According to the Pan American Health Organization, the region had reported 15,300 confirmed cases by mid-April 2026, already exceeding the 14,767 reported in all of 2025. Mexico and Guatemala accounted for the largest burdens, followed by the USA and Canada, with ongoing transmission in several countries.

Measles can be prevented with a safe, effective two-dose measles, mumps, and rubella vaccine. But, as one of the most contagious viruses known in medicine, measles can spread rapidly when population-level protection falls below the 95% threshold. The Americas was the first region in the world to eliminate endemic measles transmission in 2016, lost that status in 2018, regained it in 2024, and lost it again in 2025. In the current emergency, two-thirds of cases have no documented history of measles vaccination.

While the growth of anti-vaccine movements has been linked to outbreaks in the USA and Canada, the regional situation is a mix of mistrust, reduced access, personal and cultural beliefs, mobility, and inefficient government action. Reports from Chihuahua, Mexico, describe transmission that began in under-vaccinated Mennonite religious communities, then spread to Indigenous people, farm workers, and other populations driven by mobility and uneven access to immunisation. More than 90% of confirmed cases with known history in Mexico had no documented measles vaccination, and all confirmed deaths occurred in people without vaccination history. In Guatemala, community transmission was reported in multiple areas, with four confirmed deaths among infants younger than 1 year. The outbreak in Guatemala is being contained with a massive vaccination campaign to close a substantial immunisation gap, particularly in vulnerable, hard-to-reach groups.

About five million children in the Americas did not receive any routine vaccines between 2022 and 2024, and these gaps are concentrated particularly in peri-urban areas and remote communities and among Indigenous populations, migrants, and refugees. In Guatemala, for example, Indigenous people account for more than 40% of the population, but their limited governmental representation limits understanding of the most effective ways to operationalise public health actions across the 24 ethnic groups that inhabit the country. Scientists, clinicians, and public health agencies often assume a knowledge deficit: give people more facts, and they will make the right decision. But this approach is not effective when the messenger is not trusted, when services are hard to reach, or when messages are delivered with authority but without empathy. Vaccine hesitancy is a complex social response shaped by experience, identity, access, culture, religion, digital misinformation, and confidence in the health system.

Government accountability should be understood as an accelerant within this wider trust ecosystem. When political leaders discredit public health agencies, replace independent technical advisers with ideological loyalists, promote unsupported treatments, or frame evidence-based guidance as a threat to personal freedom, they teach the public that evidence is negotiable. The current situation in the USA shows how vaccine policy can become an instrument of political identity. The COVID-19 pandemic offered similar lessons across the region, where unsupported treatments were promoted despite weak evidence, and public health guidance was sometimes subordinated to political loyalty.

Rebuilding protection against measles and other preventable diseases will require change on multiple fronts. Countries need a reliable vaccine supply, high-quality surveillance, rapid outbreak response, and effective planning to identify who is being missed and why. But we will also need to counteract misinformation and rebuild trust in science and evidence-based medicine. Experimental evidence shows that online vaccine misinformation can reduce vaccination intention, especially when false claims borrow the language of science. This speaks to the urgent need to make social media platforms accountable for amplifying false claims about health, and to regulate artificial intelligence tools as more people resort to them for medical advice. As direct contacts and sources of knowledge, clinicians need time, special training, and tools for trust-building conversations. Interdisciplinary approaches that connect doctors with trusted community messengers, parent networks, Indigenous and local leaders, and faith-based organisations, should be prioritised and funded by governments to deliver language-adapted communication and practical access points.

The Americas achieved elimination in the past with a strong history of vaccination programmes, community engagement, and solid primary care systems. Measles resurgence revealed that our immunisation programmes are missing children, community trust is weakened, and political actors have dismissed evidence-based decisions. Regaining elimination status requires governments to place evidence, equity, and trust back at the centre of public health.

